# A retrospective review of facility-level obstetric complications and stillbirths in southern Haiti, 2013 – 2016

**DOI:** 10.26633/RPSP.2019.95

**Published:** 2019-12-09

**Authors:** Alka Dev, Keegan O’Hern, Joseph Yves Domerçant, Gerard Lucien, Lafortune Lucie, Reynold Grand-Pierre, Peter F Wright

**Affiliations:** 1 Geisel School of Medicine Dartmouth College Lebanon, New Hampshire United States of America Geisel School of Medicine, Dartmouth College, Lebanon, New Hampshire, United States of America.; 2 Maternity Department Hopital Immaculée Conception Les Cayes Haiti Maternity Department, Hopital Immaculée Conception, Les Cayes, Haiti.; 3 Department of Family Health Ministry of Health and Population Port-au-Prince Haiti Department of Family Health, Ministry of Health and Population, Port-au-Prince, Haiti.

**Keywords:** Eclampsia, dystocia, cesarean section, postpartum hemorrhage, stillbirth, Haiti, Eclampsia, distocia, cesárea, hemorragia posparto, mortinato, Haití, Eclampsia, distocia, cesárea, hemorragia pós-parto, natimorto, Haiti

## Abstract

**Objective.:**

To assess the incidence of obstetric complications—eclampsia, dystocia, cesarean section, postpartum hemorrhage, and stillbirths—in hospitals in southern Haiti in 2013 – 2016 and to discuss implications for improvements to the surveillance of birth outcomes.

**Methods.:**

This was a cross-sectional, retrospective study of data for 32 442 deliveries recorded in 2013 – 2016 by the Integrated Monitoring, Evaluation, and Surveillance System for facilities across three departments and one high-volume hospital in southern Haiti. Annual incidence rates of eclampsia, dystocia, cesarean section, postpartum hemorrhage, and stillbirths (both macerated and fresh) were calculated.

**Results.:**

The incidence of eclampsia in the study sample was 2% – 3% and of dystocia approximately 5%, comparable to elsewhere in Haiti and other low-income countries. Cesarean delivery rates averaged about 15% annually. Postpartum hemorrhage rates were lower than published data from similar settings. Stillbirth rates ranged from 30 – 62 per 1 000 births at all facilities, higher than previously recorded by the country’s population surveys. The rates of macerated stillbirths were remarkably high, close to 50% of total stillbirths, indicating severe delays in seeking or receiving emergency obstetric care.

**Conclusions.:**

This study provides important benchmarks for the current burden of preventable labor- and delivery-related complications in Haiti. Surveillance data suggest an urgent need for the management of hypertensive disorders during pregnancy, timely cesarean sections for dystocia, and management and treatment of postpartum hemorrhage in Haiti. Frequent data reviews may help address facility-specific bottlenecks.

## RESUMEN

**Objetivo.:**

Evaluar la incidencia de las complicaciones obstétricas, como la eclampsia, la distocia, las cesáreas, la hemorragia posparto y la mortinatalidad, en los hospitales del sur de Haití en el período comprendido entre los años 2013 y 2016, y analizar las implicaciones para realizar mejoras en la vigilancia de los resultados perinatales.

**Métodos.:**

Se trata de un estudio transversal y retrospectivo de los datos de 32 442 partos registrados en el período 2013-2016 mediante el sistema integrado de seguimiento, evaluación y vigilancia para establecimientos en tres departamentos y un hospital de gran actividad en el sur de Haití. Se calcularon las tasas de incidencia anuales de eclampsia, distocia, cesáreas y hemorragia posparto, así como de mortinatalidad (mortinatos macerados y frescos).

**Resultados.:**

La incidencia de eclampsia en la muestra de estudio fue de entre 2% y 3% y de distocia, de aproximadamente 5%, equiparable a cualquier lugar en Haití y otros países de ingresos bajos. Las tasas promedio de parto por cesárea se aproximaron al 15% anual. Las tasas de hemorragia posparto fueron inferiores a los datos publicados sobre entornos similares. Las tasas de mortinatalidad comprendieron entre 30 y 62 por 1 000 nacimientos en la totalidad de establecimientos, un resultado mayor al que registrado con anterioridad en las encuestas de población del país. Las tasas de mortinatos macerados fueron notablemente elevadas, cerca de 50% de la mortinatalidad total, lo que pone de manifiesto retrasos graves a la hora de solicitar u obtener atención obstétrica de urgencia.

**Conclusiones.:**

Este estudio ofrece puntos de referencia relevantes para la carga actual de complicaciones prevenibles del embarazo y el parto en Haití. Los datos de vigilancia indican que existe una urgente necesidad de tratamiento de los trastornos hipertensivos durante el embarazo, de cesáreas por distocia a tiempo, y de atención y tratamiento de la hemorragia posparto en Haití. Para abordar los obstáculos propios de los establecimientos puede ser útil realizar análisis frecuentes de los datos.

## RESUMO

**Objetivo.:**

Avaliar a incidência de complicações obstétricas – eclâmpsias, distocias, cesarianas, hemorragias pós-parto e natimortos – em hospitais na região Sul do Haiti no período de 2013 a 2016 e discutir as implicações para melhorar a vigilância dos resultados dos partos.

**Métodos.:**

Este foi um estudo transversal, retrospectivo, com dados de 32 442 partos registrados pelo Sistema Integrado de Monitoramento, Avaliação e Vigilância para estabelecimentos de saúde situados em três divisões político-administrativas (denominadas de departamentos) e um hospital de alto volume, todos situados na região Sul do Haiti, no período de 2013 a 2016. Foram calculadas as taxas anuais de incidência de eclâmpsias, distocias, cesarianas, hemorragias pós-parto e natimortos (macerados e frescos).

**Resultados.:**

Na amostra do estudo, a taxa de incidência de eclâmpsias foi de 2 a 3%, e a de partos distócicos de aproximadamente 5%, comparáveis com as de outras localidades no Haiti e com outros países de baixa renda; as cesarianas apresentaram média anual de aproximadamente 15%; as taxas de hemorragia pós-parto foram menores que as publicadas sobre estabelecimentos similares. Em todos os estabelecimentos, as taxas de partos de natimortos variaram de 30 a 62 por 1 000 nascimentos, sendo mais altas que as anteriormente registradas pelos levantamentos populacionais do país. As taxas de natimortos macerados foram excepcionalmente altas, aproximadamente 50% do total de partos de natimortos, o que indica que há graves atrasos, seja na procura por atenção obstétrica de emergência ou no recebimento dessa atenção.

**Conclusões.:**

Esse estudo fornece importantes parâmetros para determinar a atual carga de complicações passíveis de prevenção relacionadas com o trabalho de parto e com o parto propriamente dito no Haiti. Os dados de vigilância sugerem que, no Haiti, há uma necessidade urgente de manejar distúrbios hipertensivos durante a gestação, realizar cesarianas em tempo oportuno nos casos de distocias e manejar e tratar hemorragias pós-parto. Revisões frequentes dos dados podem ajudar a identificar os gargalos específicos de cada estabelecimento.

In 2015, Haiti had the highest maternal mortality rate of any country in Latin America and Caribbean (LAC), an estimated 359 maternal deaths per 100 000 livebirths (1). The country’s estimated neonatal mortality rate was 25 deaths per 1 000 livebirths compared to 9.3 / 1 000 in LAC (2), and the stillbirth rate was 25 deaths per 1 000 total births compared to 8.2 / 1 000 in LAC (3); both were also the highest in LAC.

In Haiti, only 29% of women in the country’s rural areas and 39% in urban areas deliver in a health facility (4). Given the low rates of facility births and incomplete vital registration systems, estimates for maternal and neonatal mortality and stillbirths come from statistically representative household surveys, such as the Demographic Health Survey (4). Nevertheless, case surveillance data and facility records, when available, can provide documentation of the burden of labor- and delivery-related complications among women giving birth in a health facility. Understanding the epidemiology of poor maternal health outcomes can inform resource needs for key interventions at the facility level, and contribute to improved health system planning for prevention of labor- and delivery-related complications (5, 6).

Among labor- and delivery-related complications, hemorrhage, hypertensive disorders, maternal infection, and prolonged labor cause nearly 60% of maternal deaths and contribute significantly to stillbirths and neonatal mortality (7 – 9). Preeclampsia and eclampsia are the most common hypertensive disorders that occur during pregnancy. Approximately 2% – 4% of deliveries in low- and middle-income countries are complicated by preeclampsia and 1% – 3% by eclampsia, with the highest rates occurring in the African Region (10). Published data from Haiti show preeclampsia rates as high as 7% among women delivering at a rural birthing center (11).

Data on protracted or arrested labor, also referred to as dystocia or obstructed labor, are scarce from low-income countries, but some hospital-based reviews show rates of 8% –12% in Ethiopia (12, 13), 5% in Nigeria (14), and 4% in Bangladesh (15). A study of six hospitals in Uganda showed perinatal mortality was more than double in women who experienced obstructed labor (16). Postpartum hemorrhage (PPH) is another major cause of maternal morbidity and mortality worldwide. The overall global prevalence of PPH is estimated to be 11%, ranging from 8% in LAC to 26% in Africa (17, 18).

A devastating outcome of labor and delivery complications is stillbirth. The majority of the global stillbirth burden is borne by women in resource-poor settings. The estimated average global rate is approximately 18.4 deaths per 1 000 births, with rates ranging from 1.3 / 1 000 births in Iceland to 43.1 / 1 000 births in Pakistan (3).

The objective of this study was to assess the incidence of obstetric complications—eclampsia, dystocia, cesarean section, postpartum hemorrhage, and stillbirths—in hospitals in the southern region of Haiti in 2013 – 2016 and to discuss implications for improvements to the surveillance of birth outcomes.

## MATERIALS AND METHODS

### Data and sampling

**FIGURE 1.**
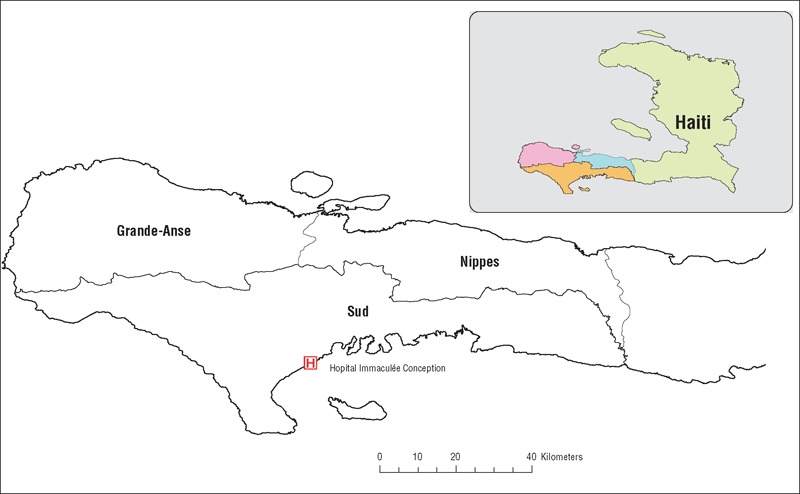
Map of Haiti showing the study area’s three departments and largest hospital, the Hopital Immaculée Conception

***Sources.*** Haiti Evaluation de la Prestation des Services de Soins de Sante 2013 (IHE and ICF International); Haiti Health Facilities Master List 2010 (MSPP).

This was a cross-sectional, retrospective study to determine incidence rates of obstetric complications and stillbirths from three departments in southern Haiti during 2013 – 2016. Data from Hopital Immaculée Conception (HIC), the area’s largest hospital, was also reviewed. Figure 1 shows the location of the Sud, Nippes, and Grand Anse departments, and HIC. Data on 32 442 deliveries was extracted for the study area’s eight hospitals from the Integrated Monitoring, Evaluation, and Surveillance System (MESI), managed by the Ministry of Public Health and Population (MSPP; 19). While MESI began as an HIV/AIDS-monitoring tool, it was expanded to include other data, including labor and delivery, from its participating facilities. As part of routine reporting, data from the maternity ward registers is aggregated on paper and then uploaded to MESI. At HIC, two nurses were responsible for these tasks— the chief maternity nurse tabulated the data on paper and an infectious disease nurse, trained in MESI, uploaded it. We assumed the arrangements were similar in other settings. MSPP checks MESI entries for errors, verifies corrections, and makes its data available on a public website.^4^

4Personal communication with Reynold Grand-Pierre, Director of Family Health, MSPP, May 2019.

To consider complete years of data, this study focused on 2013 – 2016; data from 2017 were missing for several facilities. The unit of observation was the facility, including all health centers and hospitals electronically reporting labor and delivery data to MESI. No data were reported on maternal infections.

MESI has data for over 1 000 facilities in the country’s 10 departments, but delivery data are limited to health care facilities that provide maternity services and also report to MSPP. Data completion and reporting varied significantly across departments and between public and private hospitals. To support improvements to maternal and neonatal care at HIC, this study focused on the hospital itself, the Sud department (where the hospital is located), and two other departments in the same region. For each facility in the target area, 48 separate data files were downloaded—one for each month of the 4 years—into Microsoft Excel™ (Microsoft Corp., Redmond, Washington, United States). This was combined with monthly data to form a single 4-year data sheet for each facility. These data were imported and appended to form a single dataset for all facilities and months in Stata® 15 (StataCorp, College Station, Texas, United States). As additional validation, the numbers in MESI were checked against the aggregated data paper files of the HIC. They matched.

### Outcomes

Based on the common indicators for labor and delivery reports to MESI, the study focused on five measures of maternal health: incidence rates of eclampsia, dystocia, cesarean sections, postpartum hemorrhage, and stillbirths (both macerated and fresh). Box 1 lists the clinical definition of each outcome (20 – 25). We did not find alternate definitions used in Haiti; and we did verify the definitions with the HIC maternity staff. Neither the maternity registers nor the database included information on specific clinical manifestations of severe eclampsia. While the MESI database captures dystocia, we cannot speak to differences in labor patterns (19). Measuring PPH incidence can be difficult due to the inaccuracy of measuring blood-volume loss during delivery and inadequate observation of women if they leave the hospital before being discharged (26). Finally, stillbirth designation as fresh or macerated can be helpful in distinguishing between antepartum and intrapartum complications, but the designations may not be consistent across facilities (27).

For cesarean, dystocia, eclampsia, and PPH, incidence rates were calculated as percentages, just as they are reported in published literature. However, stillbirths were calculated per 1 000 deliveries to be consistent with international reporting standards. Each indicator was calculated by quarter and year and by facility and department, but only annual rates were reported. The total denominator differed for each rate because only months that had data for a given indicator were included, even if other indicators were missing. However, the difference in denominators was < 1% across all indicators at each facility. We present annual data to ensure adequate sample sizes across each period. Stata15® was used for all calculations.

BOX 1. **DEFINITIONS OF MATERNAL AND NEONATAL COMPLICATIONS**

**Dystocia:** Refers to protracted (longer than normal) or arrested (complete cessation) labor where the fetus fails to descend despite adequate uterine contractions, often requiring instrumental or cesarean delivery (20). Dystocia is associated with increased risk of postpartum morbidity for the woman and neonatal complications for the infant.

**Preeclampsia/Eclampsia:** Pre-eclampsia is a pregnancy complication characterized by the onset of hypertension (with persistent diastolic blood pressure > 90 mm Hg) and end-organ dysfunction with or without proteinuria after 20 weeks of gestation in women whose blood pressure had been normal (21). Eclampsia refers to the convulsive manifestation of preeclampsia and one of several clinical manifestations of severe preeclampsia (22). It is characterized by seizures or coma in pre-eclamptic women.

**Postpartum hemorrhage (PPH):** An obstetric emergency that typically refers to blood loss in excess of 500 mL within 24 hours of delivery, although other criteria may be used to characterize different degrees of severity (23). The most common cause of PPH is lack of uterine contraction after delivery and retention of the placenta, which does not stop blood flow to the uterus. Other causes include uterine rupture, trauma, and provider intervention. Severe preeclampsia can also cause PPH complications.

**Stillbirth (or fetal death)**: Refers to an infant born with no signs of life (breathing, heartbeat, pulsation of umbilical cord, or definite movement of voluntary muscles) at or after 28 weeks of gestation (24). Early (20 – 27 weeks) and late (28 or more weeks) stillbirths have different causes, with intrapartum complications having a role in late stillbirths that occur at the time of delivery. Maceration, rarely discussed in high-income countries, refers to visible skin and soft tissue changes consistent with fetal death before delivery (25).

### Ethics

This study was approved by the Institutional Review Board of Dartmouth College (Study #30677), Hanover, New Hampshire, United States.

## RESULTS

### Sample characteristics

**FIGURE 2.**
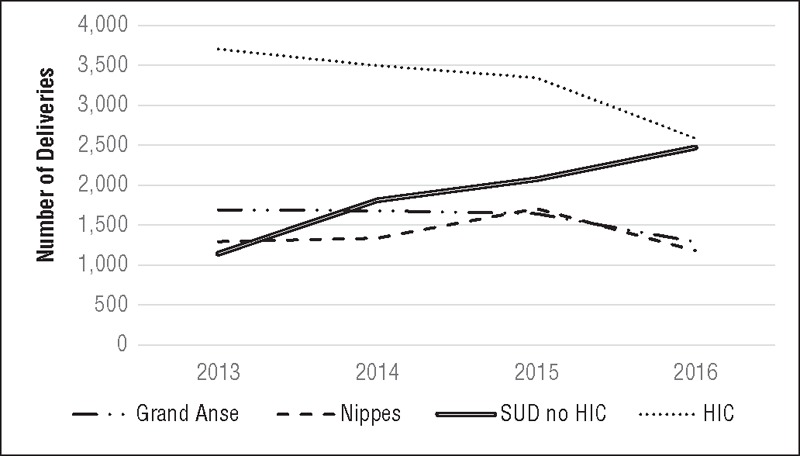
Annual number of deliveries in three departments and the area’s largest hospital, the Hopital Immaculée Conception (HIC), in southern Haiti, 2013 – 2016

***Source.*** Prepared by the authors with data from the Integrated Monitoring, Evaluation, and Surveillance System, Ministry of Public Health and Population of Haiti.

The number of deliveries was consistently higher at HIC than at other hospitals in the study area, but declined considerably in 2015 – 2016 when births at other facilities in Sud increased proportionally (Figure 2). Deliveries in Nippes and Grand Anse remained largely unchanged across the 4 years.

Regarding eclampsia, rates were below 5% for all departments; preeclampsia rates were not reported (Table 1). HIC had higher eclampsia rate in all years with the exception of 2015. In 2016, HIC reported an increase in the eclampsia rate, while the rest of Sud saw a decline; rates in Nippes and Grand Anse also rose. Dystocia rates were unchanged across the three departments and remained around 5% for all deliveries at HIC, but were higher for other facilities in Sud. Grand Anse reported unusually high rates of 15% – 20% in 2014 – 2016 (Table 1). We could not determine the association between prolonged labor and stillbirths or cesarean sections for individual women as MESI data are aggregated by facility. There was no association between rates of dystocia and cesareans at the facility level in these data (result not shown). Cesarean rates ranged from 14% – 22% and were comparable across HIC, Grand Anse, and Nippes (Table 1). Cesarean rates increased from 15% to 25% during 2014 – 2016 for the rest of Sud. There was no association at the departmental or facility level between dystocia and cesarean section rates (results not shown). PPH rates were < 4% for all three departments and at HIC (Table 1). PPH rates showed no consistent pattern for HIC, and overall rates for the departments remained unchanged over the period.

**FIGURE 3.**
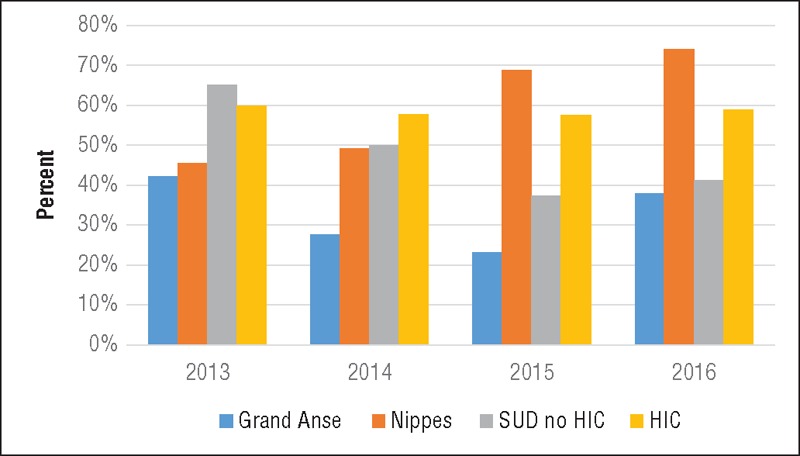
Proportion of macerated stillbirths of total stillbirths in three departments and the area’s largest hospital, the Hopital Immaculée Conception (HIC), in southern Haiti, 2013 – 2016

***Source.*** Prepared by the authors with data from the Integrated Monitoring, Evaluation, and Surveillance System, Ministry of Public Health and Population of Haiti.

Compared to the published national stillbirth rate (25 deaths per 1 000 births), the facility stillbirth rates in this study were higher (Table 1). By 2016, there were > 30 stillbirths per 1 000 births in Haiti and > 50 / 1 000 births at HIC. Proportions of macerated stillbirths were highly consistent at 60% for HIC, and from 40% – 60% at other sites (Figure 3).

## DISCUSSION

Data on more than 32 000 deliveries in three departments across southern Haiti were analyzed, while focusing on HIC where we were implementing a maternal and neonatal health project. These data serve as a baseline for the major maternal complications for deliveries at HIC—eclampsia, dystocia, cesarean section, postpartum hemorrhage, and stillbirth. We excluded data from other departments because we wanted to make comparisons and they had problematic differences in data quality and completeness. However, we did verify the accuracy of aggregate data by comparing the paper and electronic records at HIC. This was an important step in understanding how the notification process works on the facility level and showed that adding labor and delivery surveillance capacity to an existing online database is feasible, captures data without significant delay, and can be useful in tracking facility or program performance. As far as we know, this is the first study to publish facility-level data of birth outcomes in Haiti. While the results are not necessarily surprising for this setting, they highlight the value of routine surveillance of obstetrical outcomes across the country.

Fewer deliveries at HIC and increased deliveries at facilities in the rest of the Sud department did not translate into an increase in facility-deliveries overall. Therefore, the deliveries at HIC were probably being picked up by other facilities in Sud, a situation that deserves a closer look. Similarly, the increase in cesarean rates in Sud in 2014 – 2016 should be examined in relation to the increased number of deliveries at other facilities. Surveillance data can provide exactly this type of information on emerging trends and gaps in service delivery. Overall, in this sample, eclampsia rates at facilities ranged from 1% – 5% and were comparable to those reported by hospitals in similar settings. Unfortunately, eclampsia results from even higher rates of untreated preeclampsia and hypertensive disorders among pregnant women in Haiti. Detection and management of hypertensive disorders and proteinuria are essential components of routine prenatal care in Haiti, but most women do not complete the recommended four-visit schedule (4).

Dystocia rates in this sample were similar to rates reported by hospitals in other low- and middle-income countries. However, Grand Anse reported much higher rates over all 4 years. A possible reason may be the department’s low rate of facility births (27%), which may indicate that women go to a facility when labor at home does not progress well. Prolonged labor often requires instrumental or cesarean delivery, which is only available at referral hospitals in Haiti (28). While HIC has the surgical capacity to perform cesarean delivery, the availability of this procedure is affected by electrical supply, adequate anesthesia, and obstetrical coverage. Even in a hospital, labor may be further prolonged if surgery is not adequately available.

Hospital rates of cesarean sections can vary from facility to facility due to the differences in the size of the population it serves, surgical capacity, and clinical protocols (29). Any comparisons of cesarean section rates across facilities should, therefore, be made cautiously. Rates in our sample suggest that facilities with comparable delivery volume had similar rates.

**TABLE 1.** Incidence rates for major labor- and delivery-related complications in three departments and the largest hospital in southern Haiti, reported to Integrated Monitoring, Evaluation, and Surveillance System, Haiti, 2013 – 2016

Department and year	Total number of births	Eclampsia (%)	Dystocia (%)	Cesarean (%)	Postpartum hemorrhage (%)	Stillbirth rate (per 1 000 births)
Grand Anse						
2013	1 691	1	25	20	0	53.2
2014	1 680	1	18	16	1	38.7
2015	1 643	1	18	18	1	49.9
2016	1 290	2	20	21	1	57.4
Nippes						
2013	1 292	1	3	13	2	34.1
2014	1 335	3	0	13	1	48.7
2015	1 706	3	0	15	2	54.5
2016	1 179	5	0	15	1	61.9
Sud (without HIC^a^)						
2013	1 095	1	7	15	1	18.3
2014	1 810	1	8	15	1	32.0
2015	2 074	2	9	20	1	41.5
2016	2 470	2	6	25	1	36.4
HIC^a^						
2013	3 703	2	4	15	3	49.7
2014	3 497	2	4	15	2	39.2
2015	3 344	2	5	17	3	35.9
2016	2 586	4	4	14	1	54.5

a Hopital Immaculée Conception, Sud department, Haiti.

***Source.*** Prepared by the authors with data from the Integrated Monitoring, Evaluation, and Surveillance System, Ministry of Public Health and Population of Haiti.

PPH rates were lower in our sample (mostly < 4%) than in other settings (8% – 26%), and varied across the years, likely due to gaps in observation of high-risk women (17). Data from Haiti show that women stay in the hospital for an average of 2 days after childbirth (30); but in a personal communication with midwives at HIC, we learned that stays can be as short as 2 – 6 hours, even for complicated deliveries. Community-based research (30) indicates much higher PPH rates, reported as high as 10% in rural Grand Anse. Risk assessment of women likely to experience postpartum bleeding (such as lacerations, retained placenta, and abnormally adherent placenta), as well as treatment options should be part of any discharge assessment at a health facility (32).

Stillbirth rates in our facility data were twice as high as those reported by national household data (4). This was likely due to women seeking hospital care for complicated (rather than uncomplicated) pregnancies and deliveries; seeking care, but too late for effective interventions; seeking care, but not receiving appropriate and timely interventions; and due to potential under-reporting of stillbirths in household survey data. The data show high proportions of macerated stillbirths, an outcome rarely seen in high-income countries. These data suggest that strategies to prevent stillbirths are urgently needed in Haiti, that risk factors for stillbirths need to be studied further and addressed, and that interventions to reduce the time required to reach hospitals should be implemented to eliminate delay (27).

This study has several strengths. It evaluates pregnancy surveillance data from the MSPP using standardized forms across several health facilities. These data, rarely available in low-income settings, provided greater insight into the labor and delivery outcomes of health facilities at a time when facility births are being promoted across low-­income countries. The data were also verified by the maternity ward of a facility for which we are developing a comprehensive maternal and neonatal health improvement program. This built confidence in the quality of data at our intervention site. Clearly, Haitian health facilities, especially large-volume hospitals such as HIC, have to be prepared for significant maternal complications; and an evaluation of staffing and resource needs is essential for building capacity to support increased facility births. We obtained data on stillbirths, rarely reported outside of household surveys in low-income countries. Facility-level stillbirth data, particularly the macerated proportion, is immensely informative of health behaviors and outcomes for Haitian women seeking to give birth in a health care facility.

### Limitations

Limitations to our study included the lack of individual level data to assess risk factors such as age, socioeconomic status, and prior reproductive health history. We could not assess data quality for facilities other than HIC and are not certain data were consistently evaluated by MSPP for completion and accuracy. Furthermore, we could not verify which case definitions were applied across health facilities and births—definitions were assumed to be comparable across births, facilities, and settings. We also could not assess whether cesareans were medically indicated, but assumed so since Haiti is a poor country where elective cesareans are probably rare. We could not determine whether cesareans were associated with better maternal and perinatal outcomes. While it was not within the scope of our current study, we did note that public facilities were much more likely than private ones to report delivery data to MESI, which made comparisons with private facility births unfeasible. Therefore, we could not evaluate whether outcomes were better for women delivering in private or public health facilities. Finally, data were evaluated only for facilities reporting to MESI, and therefore, do not represent all deliveries in the study area nor all deliveries at health facilities and cannot be used to predict outcomes at the population level or at other health facilities.

### Conclusions

Four years of complete surveillance data on obstetrical outcomes in three departments in Haiti showed that the study area’s facilities had dystocia and eclampsia rates comparable to those of other facilities in similar settings, indicating inadequate labor monitoring and detection of complications. In addition, incidence of postpartum hemorrhage can be underestimated and difficult to compare when women are followed for < 24 hours after delivery. Discharge assessments should identify those at risk for postpartum bleeding and consider treatment options. Cesarean rates are context-dependent, but higher than average stillbirth and maceration rates in southern Haiti indicate an unmet need.

We recommend that MSPP incorporate delivery outcomes data from health care facilities into existing surveillance and notification systems to better gauge facility performance and delivery care gaps. Private sector facilities should also be required to notify these data to the national surveillance system. We also recommend that MSPP continue making the data publicly available and begin guiding facilities in using the data to set benchmarks for monitoring progress and for comparisons with other facilities.

Future research could combine surveillance information with in-person observations and interviews of women and health care providers to gain a comprehensive understanding of service delivery patterns at individual facilities in Haiti. Once the needs and current capacity of facilities have been ascertained from reliable, validated data sources, government resources and donor funds can be precisely directed at problem areas.

## Author contributions.

AD prepared the data for analysis, carried out the analysis, and wrote this article. KO assisted with data compilation and analysis. JD, GL, and LL reviewed all HIC data. RGP reviewed the manuscript and provided comments on data quality and validation. All authors read and approved the final manuscript.

## Funding.

The funders had no role in the study design, data collection or analysis, decision to publish, or preparation of the manuscript. During the time of the study, salaries for AD and PW were supported by the Children’s Prize and the WK Kellogg Foundation.

## Disclaimer.

Authors hold sole responsibility for the views expressed in the manuscript, which may not necessarily reflect the opinion or policy of the *RPSP/PAJPH* and/or PAHO.
